# Hemolytic Anemia and Pancytopenia Secondary to Vitamin B12 Deficiency: Evaluation and Clinical Significance

**DOI:** 10.7759/cureus.57286

**Published:** 2024-03-30

**Authors:** Harrison Labban, Farhana Begum, Awais Paracha, Veena John, Mohammed Islam

**Affiliations:** 1 Internal Medicine, Northwell Health, New York, USA; 2 Hematology and Medical Oncology, Northwell Health, New York, USA

**Keywords:** homocysteine metabolism, intramedullary hemolysis, pernicious-anemia, cobalamin deficiency, vitamin b12-induced hemolytic anemia

## Abstract

Severe vitamin B12 deficiency presents a diagnostic challenge due to its diverse clinical manifestations, which can mimic serious hematologic disorders such as thrombotic thrombocytopenic purpura (TTP) or leukemia. The case we present here illustrates the unique characteristics of severe B12 deficiency, highlighting key differentiators from other conditions, including decreased reticulocyte counts and markedly elevated lactate dehydrogenase levels indicative of suppressed erythropoiesis. Advanced cobalamin deficiency affects all cell lines, leading to peripheral pancytopenia. Proposed mechanisms include fragile red blood cells prone to shearing, resulting in schistocyte formation, and hyperhomocysteinemia-induced oxidative stress exacerbating hemolysis. Prompt recognition and treatment with B12 replacement are critical, as illustrated by this case of hemolytic anemia and pancytopenia secondary to pernicious anemia, to prevent severe hematologic complications.

## Introduction

Vitamin B12, a water-soluble vitamin essential for numerous cellular processes, plays a pivotal role in maintaining hematological homeostasis and neurological function. While mild B12 deficiency often presents with megaloblastic anemia and neurological symptoms resulting from demyelination, severe deficiency can manifest with unique clinical features not classically associated with the condition, such as hemolysis and pancytopenia, sometimes mimicking serious disorders like thrombotic thrombocytopenic purpura (TTP) or leukemia [1]. Understanding the intricate mechanisms underlying B12 deficiency-induced hematological abnormalities is crucial for accurate diagnosis and timely intervention.

Cobalamin’s role in DNA synthesis and hematopoiesis explains the profound results of its deficiency on erythropoiesis. Vitamin B12 acts as a cofactor for enzymes such as methionine synthase and methylmalonyl-CoA mutase, which are involved in DNA methylation and nucleotide synthesis. The unavailability of B12 inhibits DNA synthesis, effectively paralyzing red blood cell (RBC) maturation, as reflected by a low reticulocyte count - a distinguishing feature of B12-associated pancytopenia when compared to other hemolytic anemias [2].

An additional hallmark characteristic of B12-associated pancytopenia is the fragility of RBCs, which renders them susceptible to shearing and fragmentation. This fragility stems from the oxidative stress induced by B12 deficiency-including reduced reactive oxygen species scavenging, decreased glutathione levels, and impaired modulation of key cytokines-which disrupts the delicate balance of antioxidant defenses necessary for the survival of erythrocytes. As a result, RBCs become prone to mechanical damage, leading to the formation of schistocytes, a hallmark characteristic of intramedullary hemolysis observed in both B12 deficiency secondary to pernicious anemia and diet-related B12 deficiency [2,3].

In addition to its direct impact on erythropoiesis and RBC fragility, cobalamin deficiency contributes to hemolysis through secondary mechanisms, notably hyperhomocysteinemia. Elevated levels of homocysteine, a byproduct of methionine metabolism, have been implicated in endothelial damage and oxidative stress. The cytotoxic effects of hyperhomocysteinemia induce further oxidative stress within the erythrocytes, exacerbating their fragility and predisposing them to shear and fragmentation, thus perpetuating the cycle of hemolysis [4].

## Case presentation

Clinical summary

A 65-year-old male, previously healthy, was admitted to the hospital due to anemia identified in outpatient labs, reporting a recent weight loss of six pounds over two months, loss of appetite, exacerbated dyspnea on exertion, subjective fevers, nocturia, and lightheadedness. The patient is a retired postal worker who, at baseline, is physically active, including biking and lifting weights weekly. Given his excellent health, the patient did not regularly see doctors and did not know his baseline hemoglobin. He reports being in his usual state of good health before he began to experience progressively unintentional weight loss beginning two months ago, which over the past 2-3 weeks has been associated with shortness of breath and dyspnea on exertion. His primary care doctor repeated outpatient labs and identified a hemoglobin level of 5, at which time the patient was directed to report to the emergency department.

Initial vital signs were significant for sinus tachycardia. Physical examination revealed only mild scleral icterus. Laboratory findings were significant for pancytopenia with a hemoglobin level of 5.3 g/dL (mean corpuscular volume 120 fL), platelet count of 55,000/mm^3^, and WBC level of 3.21 × 10^9^/L. Hemolysis markers were elevated (LDH at 3111 U/L, decreased haptoglobin of <20 mg/dL, positive direct Coombs), while ferritin levels were normal. Despite unclear iron studies due to a recent blood transfusion by emergency department staff soon after arrival, AST was increased at 67 U/L, and total bilirubin was 1.6 mg/dL. Tumor lysis labs were negative, and CT imaging demonstrated short-axis retroperitoneal and mesenteric lymph nodes. Further testing revealed normal folate levels but severely decreased B12 levels (<150 pg/mL). Elevated levels of homocysteine (96.8 umol/L) and methylmalonic acid (15,383 mmol MMA/mmol Cr), along with positive intrinsic factor antibodies and mildly positive parietal cells antibodies, confirmed the diagnosis of pernicious anemia. Upon further discussion with the patient, it was revealed that he has cousins in Spain who also have pernicious anemia.

Treatment

The patient experienced rapid symptom improvement following the transfusion of three units of blood. Intravenous immunoglobulin was administered as a single dose for potential immune thrombocytopenic purpura but was subsequently discontinued. The patient commenced high-dose B12 injections (100 mcg daily) for a total of seven days and was then discharged with a B12 injection plan (1000 mcg weekly) outpatient. He was discharged with a stable Hgb level of 8.0 g/dL.

Outcome and follow-up

After adequate blood transfusion and four days of high-dose (100 mcg) B12 injections, the patient’s hemoglobin remained stable at 8, and he was discharged with a plan for lifelong (1000 mcg weekly) B12 injections. Appointments were arranged for three additional doses with his primary care office before transitioning to once-weekly injections. The patient was instructed to follow up with his primary care doctor one to two weeks after discharge to recheck hemoglobin levels and to present to the emergency department if he experienced shortness of breath, fatigue, or decreased appetite. Six months after admission, the patient had regained his normal appetite and returned to his baseline functional status, tolerating regular physical activity including biking and lifting weights. His B12 and hemoglobin levels remain stable at two years after discharge (628 pg/mL and 12.7 g/dL, respectively) with strong adherence to his supplementation regimen.

## Discussion

Traditional risk factors for vitamin B12 deficiency include gastric or small intestine resections, inflammatory bowel disease, prolonged use of metformin or acid-suppressing medications, strict vegetarians or vegans, and advanced age (adults over 75 years) [5]. The American Academy of Family Physicians (AAFP) does not recommend routine screening for vitamin B12 deficiency in average-risk adults; however, AAFP guidelines recognize B12 deficiency as a common cause of megaloblastic anemia, various neuropsychiatric symptoms, “and other clinical manifestations” [6]. The case we present here, and similar cases published previously, underscore the true diversity of clinical presentations of severe vitamin B12 deficiency. Keskin and Keskin highlight a 15-year-old vegetarian boy presenting with pancytopenia, icterus, and splenomegaly, mimicking acute leukemia and pseudothrombotic microangiopathy (anemia, thrombocytopenia, and schistocytosis caused by vitamin B12 deficiency). Prompt cobalamin replacement resulted in significant clinical and laboratory improvement within two months [1]. The high potential for recovery and relative simplicity of curative therapy necessitates a high degree of clinical suspicion as the signs and symptoms of the condition vary greatly. For example, in contrast to Keskin, Rao et al. present a case of pseudo-thrombotic microangiopathy in a 74-year-old man with exertional dyspnea and pancytopenia, revealing severe cobalamin deficiency from pernicious anemia. In this case, they emphasize the necessity of recognizing reticulocytopenia as a distinguishing feature of pseudo-TMA to avoid misdiagnosis as TTP and unnecessary aggressive therapy [2]. These cases collectively stress the importance of early recognition and treatment of cobalamin deficiency to prevent potentially severe hematologic complications.

## Conclusions

In summary, severe vitamin B12 deficiency poses diagnostic challenges due to its varied clinical presentations, often resembling serious hematologic disorders such as TTP and leukemia. Distinct features of cobalamin deficiency, such as low reticulocyte count and elevated lactate dehydrogenase levels, aid in differentiation and prompt intervention. Mechanisms involving inhibited erythropoiesis and oxidative stress underscore the complexity of B12 deficiency-induced hematologic abnormalities and the importance of timely treatment with B12 replacement in preventing severe hematologic complications and facilitating patient recovery.
